# Combined Effects of Instrumentation Geometry and Sealer Type on Mandibular Premolar Root Fracture Resistance

**DOI:** 10.7759/cureus.103010

**Published:** 2026-02-05

**Authors:** Funda Yilmaz, Berkuk Sayar, Berna Aslan, Mustafa A Badi

**Affiliations:** 1 Oral and Maxillofacial Radiology, Touro College of Dental Medicine, Hawthorne, USA; 2 Endodontics, Ankara University Faculty of Dentistry, Ankara, TUR; 3 Oral and Maxillofacial Radiology, Maurice H. Kornberg School of Dentistry, Temple University, Philadelphia, USA

**Keywords:** bioceramic sealer, fracture resistance, nickel-titanium files, root canal preparation, vertical root fracture

## Abstract

Aim

The aim of this study was to evaluate the effects of different root canal preparation parameters (file size and taper) and sealer types on the fracture resistance of endodontically treated mandibular premolar roots.

Methods

Ninety extracted single-rooted mandibular premolars were randomly assigned to eight experimental groups (n = 10 per group) and one control group (n = 10). Experimental groups were prepared using FKG RACE EVO nickel-titanium rotary files with sizes 25 or 30 and tapers of 0.04 or 0.06 and obturated using a single-cone technique with either TotalFill BC Sealer (bioceramic) or AH Plus (epoxy resin-based). Control specimens remained untreated. After seven days of storage at 37°C and 100% humidity, fracture resistance was measured using a universal testing machine at a crosshead speed of 1 mm/min until fracture occurred. Data were analyzed using the Kruskal-Wallis and Mann-Whitney U tests, with statistical significance set at α = 0.05.

Results

No statistically significant differences were found among the eight experimental groups (χ² = 11.64, df = 8, p = 0.076, η² = 0.045), although a small effect size was observed. Consistent trends were noted: bioceramic sealer groups demonstrated 10.7% higher mean fracture resistance (420.13 N; 89.8% of control) compared with epoxy resin-based groups (379.42 N; 81.2% of control). Size 25 preparations showed an 8.5% advantage (416.06 N; 89.0% of control) over size 30 (383.50 N; 82.0% of control), and 0.04 taper showed a 6.0% advantage (411.51 N; 88.0% of control) over 0.06 taper (388.04 N; 83.0% of control). The weakest group retained 67.7% of the control strength (316.35 N vs 467.58 N). High within-group variability (CV: 14-42%) likely precluded detection of statistical significance.

Conclusions

Within the limitations of this study, preparation parameters and sealer type did not produce statistically significant differences in fracture resistance. However, consistent directional trends favoring smaller preparations and bioceramic sealers suggest potential clinical relevance. These findings support the principles of minimally invasive endodontics.

## Introduction

Preservation of natural dentition through endodontic treatment is a fundamental goal in contemporary dentistry. However, endodontically treated teeth are at increased risk of structural failure, particularly vertical root fracture (VRF), which often necessitates extraction [1]. The etiology of VRF is multifactorial, involving both iatrogenic factors during treatment and functional stresses during clinical service [2,3].

VRF is an increasingly recognized cause of extraction in endodontically treated teeth [1,2]. Premolars are particularly vulnerable, with prevalence rates ranging from 48% to 53% among extracted premolars and accounting for approximately 52% of all VRF cases in endodontically treated teeth [4,5]. Mandibular premolars specifically demonstrate high susceptibility, with 48-53% of extractions attributed to VRF [4,6]. This vulnerability is related to their narrow mesiodistal dimension and oval root canal morphology, anatomical features that concentrate stress during both endodontic instrumentation and functional loading.

Root canal preparation inherently compromises tooth structure by removing infected dentin and creating adequate space for irrigation and obturation. The extent of structural compromise depends on multiple factors, including preparation size (apical diameter), taper (coronal flaring), and instrumentation technique [7,8]. Modern nickel-titanium (NiTi) rotary systems offer a variety of size and taper combinations, yet the optimal balance between effective cleaning and preservation of structural integrity remains controversial [9,10].

The role of root canal sealers in reinforcing weakened roots has generated considerable research interest. Traditional epoxy resin-based sealers, such as AH Plus, demonstrate excellent sealing properties and long-term clinical success [11,12]. The recent introduction of calcium silicate-based bioceramic sealers has sparked debate regarding their potential to enhance fracture resistance through chemical bonding and hydroxyapatite formation [13]. In vitro evaluations of obturation materials indicate material-dependent effects on root integrity and filling adaptation, which contextualize the present comparison of TotalFill BC and AH Plus [14].

Previous investigations examining the effects of preparation parameters and sealer type on fracture resistance have yielded inconsistent findings. Some studies report that increased taper significantly reduces fracture resistance, whereas others found no significant effect [15-18]. Similarly, comparisons of bioceramic versus epoxy resin-based sealers have shown conflicting results regarding reinforcement potential [18]. These discrepancies may reflect methodological differences, variation in tooth types, or inherent biological variability.

Given the clinical significance of root fracture and the lack of consensus in the literature, this study aimed to evaluate the combined effects of preparation parameters (size 25 vs. 30; taper 0.04 vs. 0.06) and sealer type (bioceramic vs. epoxy resin-based) on the fracture resistance of endodontically treated mandibular premolar roots using a contemporary NiTi rotary system (FKG RACE EVO) that provides multiple size-taper combinations within a standardized file design.

Instrumentation geometry refers to the apical preparation size (ISO diameter) and taper (rate of canal enlargement) achieved using a single rotary file system, representing key parameters that determine the final canal configuration and remaining dentin thickness. The selected parameters (sizes 25-30, tapers 0.04-0.06) reflect clinically relevant preparation thresholds for mandibular premolars, balancing adequate debridement with conservative dentin preservation. Given the narrow mesiodistal dimensions and oval canal morphology of mandibular premolars, these preparation sizes minimize excessive dentin removal while achieving sufficient cleaning and shaping, consistent with contemporary minimally invasive endodontic principles [9,10]. The null hypothesis was that neither preparation parameters nor sealer type would significantly affect fracture resistance.

## Materials and methods

Sample selection, inclusion/exclusion criteria, and standardization

A total of 146 recently extracted, intact human mandibular premolars were initially collected. Following extraction, teeth were stored at +4°C in saline solution containing 0.1% thymol for a maximum of three months prior to testing. Throughout all experimental procedures, specimens were kept hydrated in saline to prevent dentin dehydration and maintain mechanical properties. After applying predefined inclusion and exclusion criteria, 90 teeth were ultimately included in the study.

Teeth were excluded if they exhibited caries, cracks, previous endodontic treatment, or anatomical variations. Specimens were examined under an operating microscope (Carl Zeiss Microscopy GmbH, Munich, Germany) at ×12 magnification. Unsuitable specimens were excluded, and radiographic examination was performed to confirm single-canal anatomy, absence of external or internal root resorption, and straight root configuration.

Root dimensions were further standardized using digital calipers at 5 mm from the apex, and only teeth within the accepted range (5.21 ± 0.5 mm buccolingually and 4.76 ± 0.4 mm mesiodistally) were included. To standardize root length, teeth were measured and marked at 13 mm from the apex using an electronic digital caliper, and crowns were removed under water cooling with a precision cutting device (Micracut Precision Cutter, Metkon Instruments Inc., Bursa, Turkey).

**Figure 1:**
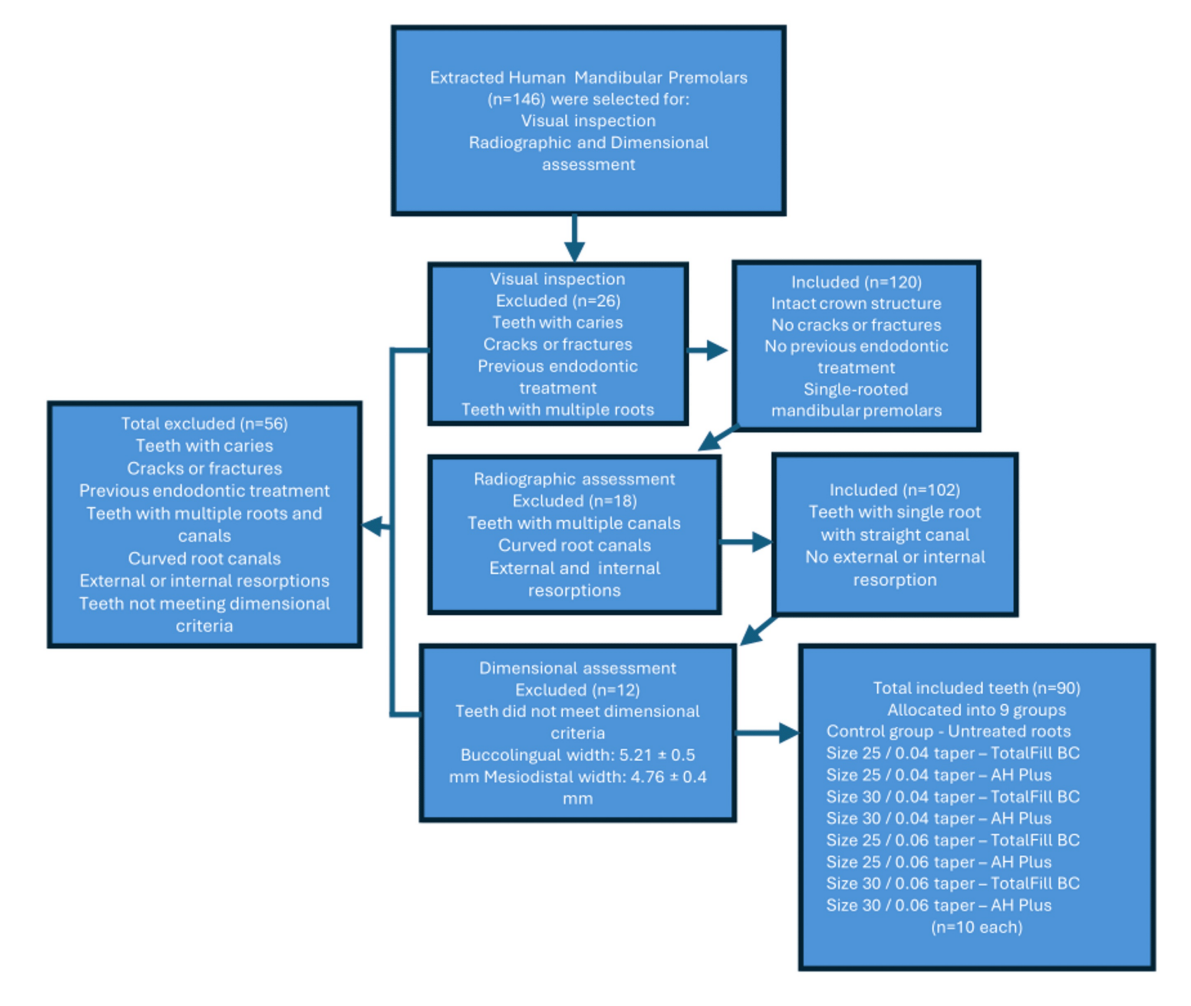
Flowchart illustrating the selection of extracted human mandibular premolars based on predefined exclusion criteria, from initial screening to final inclusion in the study

The process of sample selection, application of exclusion criteria, and final allocation of teeth into experimental groups is summarized in Figure 1.

Group allocation and root canal instrumentation

Specimens were randomly assigned using computer-generated allocation to nine groups (n = 10): one control group (untreated) and eight experimental groups based on preparation size (25 or 30), taper (0.04 or 0.06), and sealer type (TotalFill BC Sealer or AH Plus).

Working length was established 1 mm short of the apical foramen using a size 10 K-file. Glide path preparation was completed with size 15 and 20 K-files. Root canals were prepared using FKG RACE EVO NiTi rotary files (FKG Dentaire, La Chaux-de-Fonds, Switzerland) in a crown-down sequence according to the manufacturer’s specifications, at 600 rpm and 1.5 N·cm torque, to the assigned final size and taper (25/0.04, 25/0.06, 30/0.04, or 30/0.06).

Between each file, canals were irrigated with 5 mL of 2.5% sodium hypochlorite using a 30-gauge side-vented needle. Final irrigation consisted of 5 mL of 2.5% sodium hypochlorite, followed by 5 mL of 17% EDTA for three minutes [19], and 5 mL of distilled water. Canals were dried with paper points corresponding to the final preparation size.

Obturation procedure

Canals were obturated using the single-cone technique with master gutta-percha cones (Dentsply Maillefer, Ballaigues, Switzerland) matching the final preparation size and taper. Two sealers were used according to group assignment: TotalFill BC Sealer (FKG Dentaire, bioceramic) or AH Plus (epoxy resin-based; Dentsply Sirona, Charlotte, NC, USA), applied according to the manufacturers’ instructions.

For the TotalFill BC Sealer group, sealer was introduced into the root canal using its intracanal tip up to the coronal one-third of the canal. A master gutta-percha cone was coated with sealer and gently inserted to the working length. For the AH Plus group, sealer was mixed according to the manufacturer’s instructions, and a master gutta-percha cone was coated with the sealer and gently inserted to the working length.

In all groups, a master gutta-percha cone with confirmed tug-back at the working length was used before sealer application. Excess gutta-percha was removed at the canal orifice with a heated instrument, and access cavities were sealed with temporary filling material. Periapical radiographs were taken in mesiodistal and buccolingual directions to verify the quality and completeness of the root canal filling. All endodontic procedures were performed by a single trained operator, who was calibrated in advance for all instrumentation and obturation protocols to ensure procedural consistency.

Before mechanical testing, roots were embedded in self-curing acrylic resin within cylindrical molds, leaving 9 mm of the coronal portion exposed above the resin and 4 mm embedded apically to simulate clinical conditions. A 0.2 mm-thick polyether impression material was applied around the embedded portion to simulate the periodontal ligament [20]. Specimens were stored at 37°C and 100% humidity for seven days to allow complete sealer setting.

Mechanical testing

**Figure 2:**
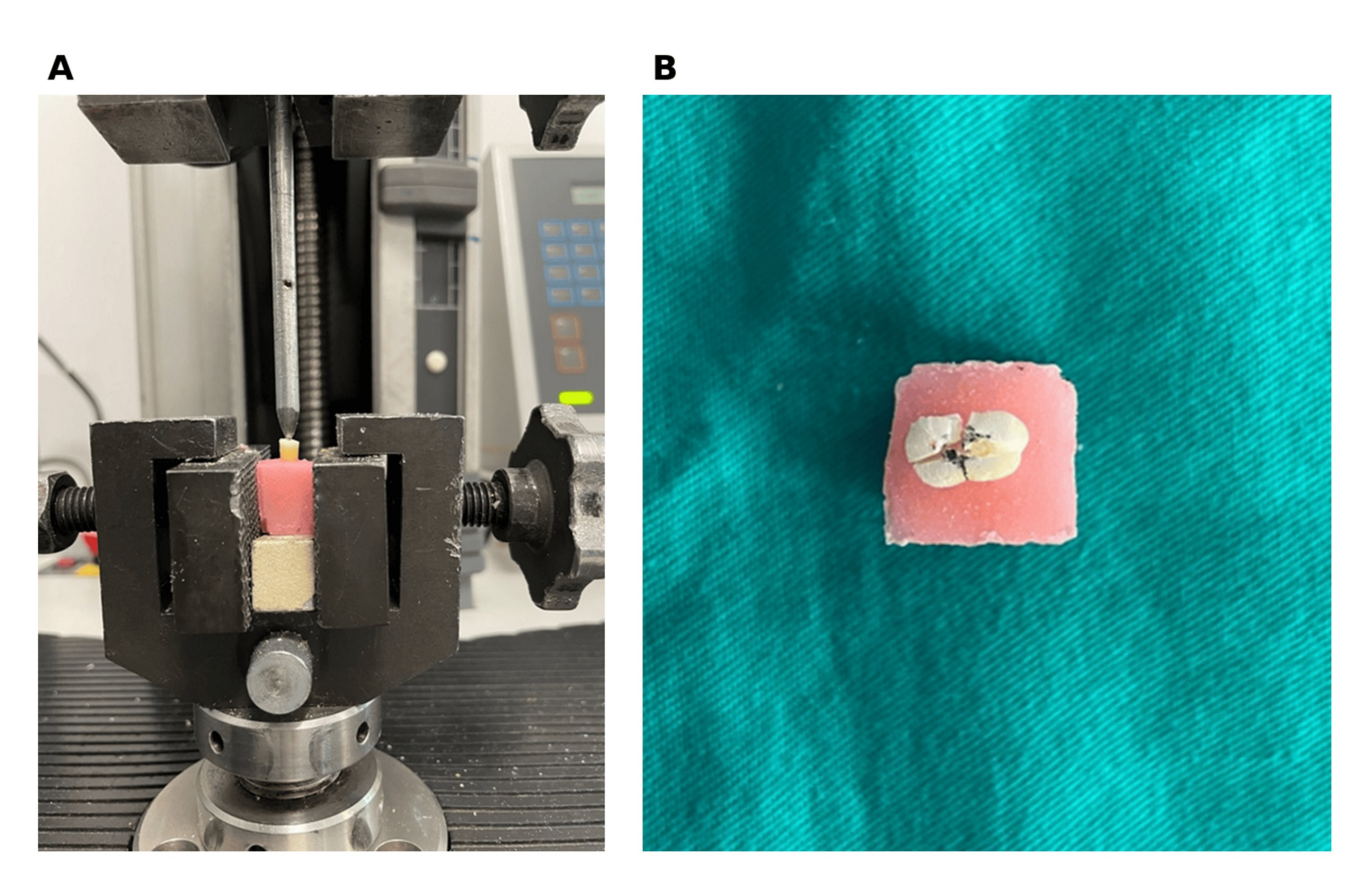
Experimental setup and representative fracture pattern (A) Specimen mounted in an acrylic resin block positioned in a universal testing machine (Instron Model 3345) with a stainless steel spherical indenter (5 mm diameter) applied at the canal orifice. The coronal 9 mm of root structure is exposed above the acrylic resin to simulate clinical conditions. (B) Representative fractured specimen demonstrating VRF extending from the canal orifice through the root length following compressive loading. VRF, vertical root fracture

Fracture resistance was measured using a universal testing machine (Model 3345; Instron, Norwood, MA, USA). A stainless steel spherical indenter (5 mm diameter) applied a vertical compressive force to the canal orifice at 1 mm/min until fracture occurred. Fracture was defined as the point at which a sudden drop in load was detected (Figure 2). Maximum load at fracture was recorded in newtons. The operator was blinded to group allocation during fracture resistance testing and data analysis.

Ethical approval

Ethical approval for this study was obtained from the Ankara University Ethics Committee (approval 18/11). Informed consent was waived by the institutional ethics committee because the study used anonymized extracted human teeth collected for routine clinical purposes, with no patient identifiers or clinical data involved.

Statistical analysis

Data were analyzed using IBM SPSS Statistics for Windows, Version 20.0 (Released 2011; IBM Corp., Armonk, NY, USA). The Shapiro-Wilk test was used to assess the normality of the distribution. Due to nonnormal distribution in several groups, nonparametric tests were employed. The Kruskal-Wallis H test was used to compare fracture resistance among all groups, with effect size calculated as eta-squared (η²). The Mann-Whitney U test was used to compare specific group pairs. Statistical significance was set at α = 0.05.

## Results

Fracture resistance values across all groups ranged from 316.35 to 467.58 N, corresponding to 67.7%-100% of the control strength. The control group demonstrated the highest mean fracture resistance (467.58 ± 65.65 N; 100% reference). Among the experimental groups, the 25/0.06 TotalFill BC Sealer group showed the highest resistance (439.51 ± 132.23 N; 94.0% of control), whereas the 30/0.06 AH Plus group exhibited the lowest resistance (316.35 ± 132.63 N; 67.7% of control). The 38.9% difference between the strongest and weakest experimental groups represents a substantial variation in fracture resistance, despite the statistical findings described below.

The Shapiro-Wilk test indicated nonnormal distribution of data (p < 0.05 for multiple groups), justifying the use of nonparametric statistical analyses. The Kruskal-Wallis H test revealed no statistically significant difference in fracture resistance among the eight experimental groups (χ² = 11.64, df = 8, p = 0.076). However, the p-value approached the conventional significance threshold (α = 0.05), and the effect size (η² = 0.045) indicated a small but potentially clinically relevant effect.

**Figure 3:**
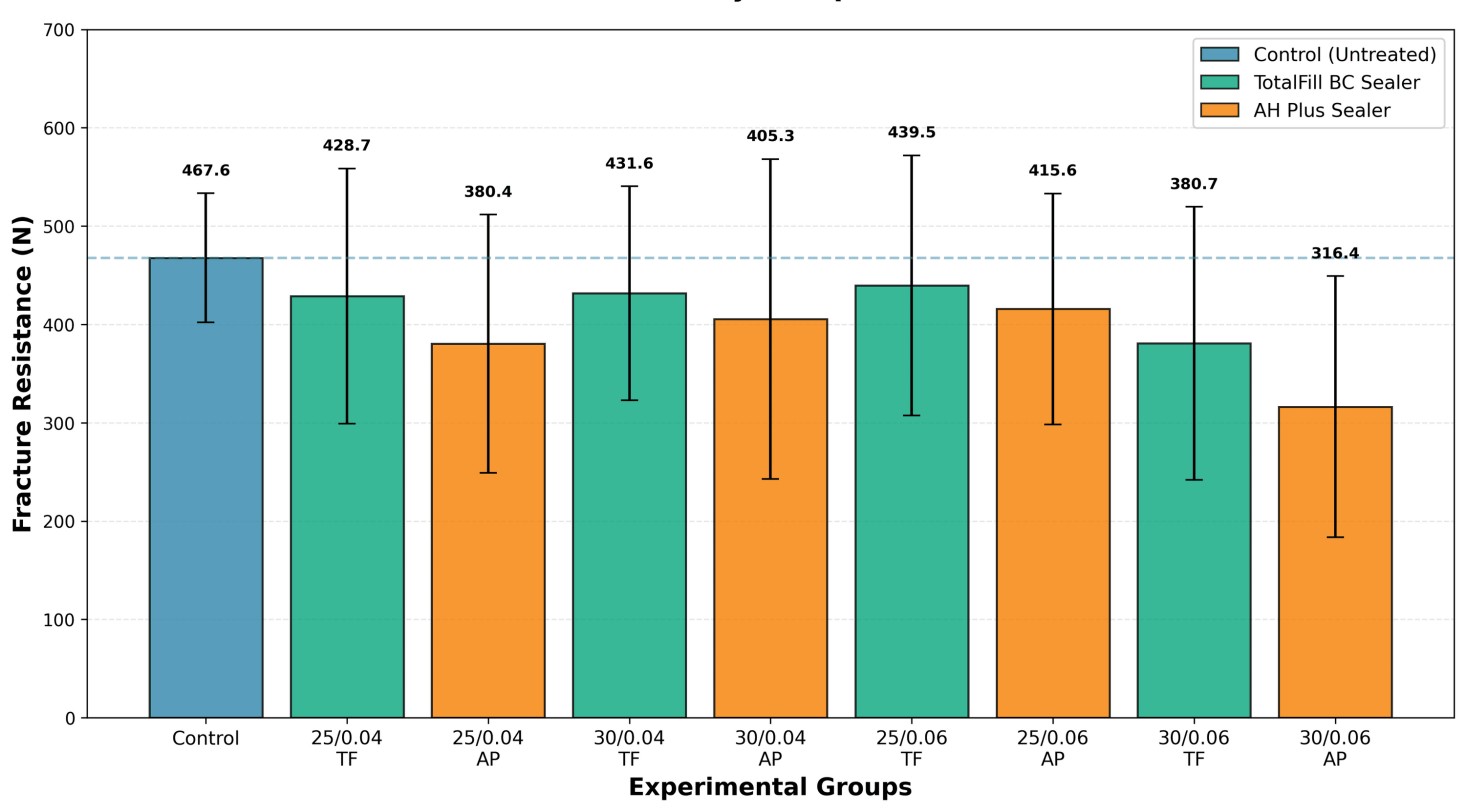
Mean fracture resistance by group with SD Mean fracture resistance (N) for control and experimental groups. Error bars represent SD. AP = AH Plus; N = newtons (unit of fracture load); TF = TotalFill BC Sealer

Descriptive statistics for all groups are presented in Table 1, and comparative data are illustrated in Figure 3.

**Table 1:** Descriptive statistics of fracture resistance Values are presented as mean ± SD, with 95% CIs, minimum, and maximum values expressed in newtons (N). The “% of control” column represents the mean fracture resistance of each experimental group relative to the control group (control = 100%). Sample size per group was n = 10 teeth. AP = AH Plus; N = newtons (unit of fracture load); TF = TotalFill BC Sealer

Group	Mean ± SD (N)	95% CI (N)	Minimum (N)	Maximum (N)	% of control
Control	467.58 ± 65.65	420.64-514.58	341.97	551.85	100
25/0.04 TF	428.74 ± 129.71	335.95-521.54	294.97	637.8	91.7
25/0.04 AP	380.36 ± 131.25	286.47-474.26	209.48	597.94	81.3
30/0.04 TF	431.60 ± 108.82	353.75-509.45	322.22	645.3	92.3
30/0.04 AP	405.34 ± 162.76	288.91-521.77	216.83	641.9	86.7
25/0.06 TF	439.51 ± 132.23	344.93-534.11	178.98	677.52	94
25/0.06 AP	415.63 ± 117.54	331.55-499.72	256.21	606.01	88.9
30/0.06 TF	380.69 ± 138.85	281.36-480.02	263.01	696.78	81.4
30/0.06 AP	316.35 ± 132.63	221.48-411.24	133.21	553.55	67.7

When data were pooled across all preparation configurations, a consistent trend emerged favoring bioceramic sealers. TotalFill BC Sealer groups demonstrated a mean fracture resistance of 420.13 ± 126.14 N (89.8% of control, n = 40 teeth), compared with 379.42 ± 135.96 N (81.2% of control, n = 40 teeth) for AH Plus groups. This represented a 10.7% advantage for bioceramic sealers (mean difference: 40.72 N), a trend that remained consistent across all four preparation combinations (size 25 or 30; taper 0.04 or 0.06). Although Mann-Whitney U testing did not indicate statistical significance (p > 0.05), the consistent directional effect across multiple subgroups suggests potential clinical relevance.

CI analysis further characterized these comparisons. TotalFill BC Sealer groups had a mean fracture resistance of 420.13 N (95% CI: 379.23-461.04 N) compared with 379.42 N (95% CI: 335.59-423.25 N) for AH Plus groups, yielding a mean difference of 40.72 N (95% CI: -18.29 to 99.72 N). The CI for this difference crossed zero, consistent with the absence of statistical significance (p = 0.076), while indicating a plausible effect size range from an 18.29 N advantage for AH Plus to a 99.72 N advantage for TotalFill BC.

Analysis of preparation parameters revealed additional trends. When pooled by size, preparations with size 25 files (n = 40) showed a mean fracture resistance of 416.06 N (89.0% of control), representing an 8.5% advantage over size 30 preparations (mean: 383.50 N; 82.0% of control, n = 40). Similarly, when grouped by taper, 0.04 taper preparations (n = 40) demonstrated a mean of 411.51 N (88.0% of control), representing a 6.0% advantage compared with 0.06 taper preparations (mean: 388.04 N; 83.0% of control, n = 40). Neither preparation size nor taper produced statistically significant effects when analyzed independently (p > 0.05 for both), suggesting these factors may have modest individual effects that become more apparent when combined with sealer selection.

Considerable within-group variability was observed across all experimental groups. Coefficients of variation ranged from 14.0% in the control group to 41.9% in the 30/0.06 AH Plus group, with experimental groups displaying coefficients between 25.2% and 41.9%. This substantial variability, consistent with the biological heterogeneity inherent to extracted human teeth, likely obscured the detection of smaller but potentially consistent treatment effects and contributed to the lack of statistical significance despite observable trends in mean values and the relatively large sample size (n = 10 per group).

## Discussion

Endodontically treated teeth are inherently susceptible to VRF due to cumulative loss of tooth structure from caries, trauma, access preparation, and canal instrumentation [1,2]. VRFs are a major cause of post-treatment tooth extraction, with mandibular premolars being particularly vulnerable [7,8]. In this study, single-rooted, single-canal human mandibular premolars were selected to minimize anatomical and biological variations and because they exhibit a high incidence of fracture following endodontic treatment [1,4,5].

The present study evaluated the combined effects of preparation parameters (size and taper) and sealer type on fracture resistance of endodontically treated mandibular premolar roots. Although the Kruskal-Wallis test did not reveal statistically significant differences among groups (p = 0.076), numerical differences were observed, warranting careful interpretation.

The lack of statistical significance does not imply an absence of biological or clinical effects. High within-group variability (CV: 14-42%) likely obscured detection of smaller but consistent differences, reflecting the biological heterogeneity inherent to extracted human teeth [12,13]. The p-value approached conventional significance thresholds, and the effect size (η² = 0.045) suggests practical relevance despite statistical nonsignificance. When evaluating negative results, it is important to consider effect sizes and consistent trends across multiple comparisons for clinical interpretation.

The 95% CI for the sealer comparison (-18.29 to 99.72 N) provides additional interpretive context beyond the p-value. While the interval crosses zero, confirming statistical nonsignificance, the point estimate of 40.72 N (a 10.7% advantage for bioceramic sealers) and the asymmetric confidence bounds suggest a directional trend of potential clinical interest. The lower bound indicates that, in the least favorable scenario, AH Plus could provide 18.3 N greater resistance, while the upper bound suggests TotalFill BC could provide up to a 99.7 N advantage. This range of approximately 118 N reflects the substantial within-group biological variability observed (coefficients of variation: 14-42%).

A notable finding was the consistent 10.7% higher fracture resistance in bioceramic sealer groups (420.13 N; 89.8% of control) compared with epoxy resin-based groups (379.42 N; 81.2% of control) across all preparation configurations. This aligns with the theoretical advantages of calcium silicate-based sealers [21]. Calcium silicate materials produce hydroxyapatite crystals, which may form chemical bonds with root dentin [21,22]. The use of TotalFill BC Sealer combined with single-cone obturation in this study aligns with recent evidence demonstrating superior fracture resistance with this specific combination [23,24]. Al-Hiyasat et al. [23] reported that TotalFill BC Sealer with a single-cone technique provided the highest fracture resistance among various sealer-technique combinations tested in mandibular premolars. This finding was corroborated by Song et al. [24], who confirmed that bioceramic sealers with a single-cone technique yielded superior fracture strength compared with resin-based sealers and alternative obturation methods.

In addition, bioceramic sealers, when used with a hydraulic condensation technique, flow into canal irregularities without applying wedging forces and can enhance the fracture resistance of roots, unlike traditional resin-based sealers [13,21,22]. The hydraulic condensation technique used with bioceramic sealers likely contributed to the observed fracture resistance outcomes. Unlike traditional compaction methods that apply significant wedging forces, hydraulic condensation distributes the sealer via fluid pressure without generating mechanical stress on the root. Studies have shown that bioceramic sealers with hydraulic condensation yield significantly higher fracture resistance compared with other obturation systems by eliminating condensation-related forces while providing chemical bonding through hydroxyapatite formation, potentially compensating for structural loss during preparation [13,21,22,25]. Previous studies have reported conflicting results [23,26], likely reflecting methodological differences and inherent biological variability.

Preparation parameters showed modest trends. Size 25 preparations demonstrated 8.5% higher resistance (416.06 N; 89.0% of control) than size 30 (383.50 N; 82.1% of control), and 0.04 taper preparations showed 6.0% higher resistance (411.51 N; 88.0% of control) than 0.06 taper. These trends are consistent with biomechanical principles, whereby greater dentin removal weakens root structure, although the lack of statistical significance indicates variability across specimens. Literature findings remain contradictory [13-16], reflecting differences in file systems, tooth anatomy, and testing conditions. Recent investigations using different file systems corroborate the protective effect of smaller tapers. Bapna et al. [27] demonstrated progressive reductions in fracture resistance with increasing taper in mandibular premolars (0.04 taper: 314.6 N, 60.7% of control; 0.06 taper: 276.7 N, 53.4% of control; 0.10 taper: 154 N, 29.7% of control; control: 518.1 N, 100%). Similarly, Shyma et al. [28], using CBCT analysis, showed that larger tapers compromise preservation of pericervical dentin, a critical determinant of fracture resistance.

The instrumentation protocol employed in this study utilized the FKG RACE EVO system with conservative preparation parameters (sizes 25-30, tapers 0.04-0.06), consistent with contemporary minimally invasive endodontic philosophy. The use of a single file system across all groups eliminated potential confounding from differing file geometries or metallurgical properties, allowing isolation of preparation parameter effects [27,28]. Recent systematic reviews support this minimally invasive approach. Usta et al. [29] concluded that smaller preparation dimensions may enhance fracture resistance, although considerable methodological heterogeneity among studies limits definitive conclusions. Puleio et al. [30], in a systematic review specifically addressing low-taper preparations, similarly reported evidence favoring reduced taper for fracture resistance, while noting the need for more rigorous comparative studies. These analyses reinforce the clinical rationale for conservative instrumentation strategies that balance adequate debridement with preservation of structural integrity.

The weakest experimental group (30/0.06 AH Plus) retained 67.7% of control strength (316.35 vs. 467.58 N), yet absolute values (316-440 N; 67.7%-94.0% of control) remained within clinically functional ranges for premolar masticatory forces [31]. However, the 38.9% variation between the strongest and weakest groups suggests that larger preparation dimensions with epoxy resin-based sealers may pose a higher fracture risk, particularly in teeth with compromised ferrules [32] or heavy occlusion. These findings support minimally invasive endodontic principles [9,10].

The relatively lower fracture resistance in AH Plus groups reflects fundamental material properties. Unlike bioceramic sealers, which chemically bond to dentin via hydroxyapatite formation, AH Plus achieves only mechanical adhesion through micromechanical interlocking with dentin collagen, providing no structural reinforcement [33,34]. Epoxy resin-based sealers lack bioactivity and biomineralization capability, explaining why they do not enhance root fracture resistance compared with unfilled controls [33,34]. The rigid, brittle nature of cured epoxy resins may concentrate rather than distribute stress [34], and the absence of dimensional expansion prevents compensation for structural loss during preparation. In contrast, bioceramic sealers are hydrophilic and insoluble, allowing them to utilize the moisture present in dentinal tubules to initiate and complete their setting reaction [21,24].

Study limitations include biological variability despite standardization efforts, in vitro conditions that do not fully replicate the oral environment, and potential EDTA-induced microhardness reduction [35] (although all groups received identical treatment). Sample size (n = 10 per group; n = 90 total) provided 60-80% power to detect medium effect sizes. However, to reliably detect the small effect size observed (η² = 0.045), larger sample sizes (n = 20-25 per group) would be required. Additional limitations include the use of single-load-to-failure testing rather than cyclic loading protocols, which would better simulate clinical masticatory conditions.

Future research should address several key areas. First, larger sample sizes (n = 20-25 per group) are needed to adequately detect the small-to-medium effect sizes observed. Second, cyclic loading protocols would better simulate clinical masticatory conditions and may reveal differences not apparent under single-load testing. Third, investigation of restored versus nonrestored specimens would clarify the interaction between endodontic treatment and coronal restoration on fracture resistance. Given emerging evidence from recent systematic reviews [26,28], future studies should specifically compare minimally invasive preparation protocols (≤0.04 taper) with conventional larger tapers (≥0.06) using standardized methodology to establish clinical thresholds for fracture risk. Finally, clinical studies with adequate follow-up periods remain necessary to validate laboratory findings and determine the actual clinical significance of the observed trends.

## Conclusions

Within the limitations of this in vitro study, the findings emphasize the importance of conservative endodontic strategies for preserving the structural integrity of teeth. Selection of appropriate preparation dimensions, combined with modern bioceramic sealers, may help maintain root strength and reduce the risk of fracture. These results underscore the clinical value of minimally invasive instrumentation and careful material selection in endodontic practice. Further research, particularly under conditions that simulate long-term functional stresses, is warranted to better define optimal preparation and obturation approaches for enhancing fracture resistance and improving treatment outcomes.
